# Online LI-rTMS during a Visual Learning Task: Differential Impacts on Visual Circuit and Behavioral Plasticity in Adult Ephrin-A2A5^–/–^ Mice

**DOI:** 10.1523/ENEURO.0163-17.2018

**Published:** 2018-02-14

**Authors:** Eugenia Z. Poh, Alan R. Harvey, Kalina Makowiecki, Jennifer Rodger

**Affiliations:** 1 School of Biological Sciences, The University of Western Australia, Crawley, WA 6009, Australia; 2 School of Human Sciences, The University of Western Australia, Crawley, WA 6009, Australia; 3 Perron Institute for Neurological and Translational Research, Nedlands, WA 6009, Australia; 4 Department of Systems Neuroscience, JFB Institute for Zoology and Anthropology, University of Goettingen, Goettingen, 37075, Germany

**Keywords:** Brain stimulation, motivation, plasticity, rTMS, visual system

## Abstract

Repetitive transcranial magnetic stimulation (rTMS) induces plasticity in normal and abnormal neural circuitries, an effect that may be influenced by intrinsic brain activity during treatment. Here, we study potential synergistic effects between low-intensity rTMS (LI-rTMS) and concurrent neural activity in promoting circuit reorganization and enhancing visual behavior. We used ephrin-A2A5^–/–^ mice, which are known to possess visuotopic mapping errors that are ameliorated by LI-rTMS, and assessed the impact of stimulation when mice were engaged in a visual learning task. A detachable coil was affixed to each mouse, and animals underwent 2 wk of 10-min daily training in a two-choice visual discrimination task with concurrent LI-rTMS or sham stimulation. No-task controls (+LI-rTMS/sham) were placed in the task arena without visual task training. At the end of the experiment, visuomotor tracking behavior was assessed, and corticotectal and geniculocortical pathway organization was mapped by injections of fluorescent tracers into the primary visual cortex. Consistent with previous results, LI-rTMS alone improved geniculocortical and corticotectal topography, but combining LI-rTMS with the visual learning task prevented beneficial corticotectal reorganization and had no additional effect on geniculocortical topography or visuomotor tracking performance. Unexpectedly, there was a significant increase in the total number of trials completed by task + LI-rTMS mice in the visual learning task. Comparison with wild-type mice revealed that ephrin-A2A5^–/–^ mice had reduced accuracy and response rates, suggesting a goal-directed behavioral deficit, which was improved by LI-rTMS. Our results suggest that concurrent brain activity during behavior interacts with LI-rTMS, altering behavior and different visual circuits in an abnormal system.

## Significance Statement

Noninvasive brain stimulation effects may depend on brain activity at the time of treatment. We have developed a method to investigate the impact of “online” low-intensity repetitive transcranial magnetic stimulation (LI-rTMS) on behavior, enabling elucidation of the anatomic and behavioral effects of rTMS for more effective translation into the clinic. Here we delivered LI-rTMS to transgenic mice while they were undertaking a visual learning task. Our results suggest that concurrent brain activity interacts with LI-rTMS, resulting in different outcomes in different projections. Unexpectedly, we found goal-directed behavioral deficits in ephrin-A2A5^–/–^ mice that were partially rescued by LI-rTMS.

## Introduction

Accurate cytoarchitectonic development of gray matter and establishment of appropriately organized synaptic circuitries within and between brain regions are elements required for normal, healthy brain function. Although the capacity for beneficial, large-scale reorganization is limited in adulthood (Hensch, 2005; Takesian and Hensch, 2013), some degree of plasticity is retained throughout life, allowing subtle changes in structure, connectivity, and function that underpin learning and memory. Tools that enhance plasticity capabilities show promise in facilitating plasticity for functional repair, particularly as an adjuvant treatment, and have been explored not only in healthy and normally aging subjects, but also in patients with neurologic and psychiatric disorders associated with abnormal neural circuit organization and dysfunctional plasticity processes (Krucoff et al., 2016).

Repetitive transcranial magnetic stimulation (rTMS), a noninvasive brain stimulation (NBS) technique used to target and modulate the excitability of specific brain regions, has been extensively studied for the treatment of various neurologic and psychiatric conditions (Lefaucheur et al., 2014). Importantly, the effects of rTMS can outlast the period of stimulation (Pell et al., 2011). However, because NBS modulates neuronal activity, outcomes may depend on the individual’s brain activity during rTMS application (Ridding and Ziemann, 2010).

Studies have shown that rTMS has stronger effects in humans engaged in a behavioral task or receiving sensory input, compared with those at rest. For example, in healthy volunteers, 5 Hz rTMS combined with tactile coactivation enhanced tactile discrimination (Ragert et al., 2003), and combination with a motor learning task increased rate of skill acquisition (Narayana et al., 2014). Likewise, rTMS during maximal voluntary hand contraction enhanced and prolonged increases in motor cortical excitability compared with rTMS alone (Yin et al., 2015). Similarly, in stroke patients, engaging in motor practice during rTMS increased intracortical facilitation and improved performance in a range of motor tests (box and block test, force steadiness), but practice or rTMS alone improved only force steadiness, with no change in cortical excitability (Massie et al., 2017). The implication is that neural activity evoked by either rTMS or training alone may be insufficient to induce lasting plastic changes, but these interventions may be additive and increase capability for task-specific activity-dependent plasticity (Wessel et al., 2015).

We previously showed that 2 wk daily low-intensity rTMS (LI-rTMS, strength approximately 2 orders of magnitude lower than the high-intensity magnetic fields used clinically) improves abnormal visual circuit topography and repairs abnormal optomotor reflexes (head tracking performance) in adult ephrin-A2 and -A5 knockout (ephrin-A2A5^–/–^) mice without disrupting normal connectivity (Rodger et al., 2012; Makowiecki et al., 2014). Despite these improvements, about half of the abnormal retinotectal projections persist following LI-rTMS (Rodger et al., 2012), and primary visual cortex (V1) afferent and efferent projections, although more accurate, remain more disorganized compared with control wild-type mice (Makowiecki et al., 2014).

Given mounting evidence that rTMS interacts with activity evoked by sensory input/behavioral tasks, we used the adult ephrin-A2A5^–/–^ mouse model to investigate whether LI-rTMS during a targeted visual learning task would further improve topographic reorganization of V1 afferent and efferent projections compared with LI-rTMS alone. We used a two-choice visual discrimination task because acquisition and discrimination between visual stimuli reliably alters V1 activity, changing V1 neuron response coding, spine density, and inhibition-excitation balance (Wang et al., 2013). Additionally, learning to discriminate between visual stimuli alters V1 network interactions (Kamiyama et al., 2016), with feed-forward perceptual changes (e.g., acuity detection of patterned visual stimuli) subserved by the geniculocortical pathway (Hembrook-Short et al., 2017) and top-down connectivity changes between V1 and subcortical regions, including the superior colliculus (SC), linked to gaze and attentional shifts, target selection, and reward-directed action (Schall et al., 2011; Gottlieb, 2012). We assessed the effects of LI-rTMS alone or combined with the task on visual system topography, both anatomically (corticotectal and geniculocortical projections) and functionally (head tracking in the visuomotor task). In addition, because there is evidence from human studies that rTMS may enhance cognition and modulate motivation and reward-seeking behavior (Grall-Bronnec and Sauvaget, 2014; Protasio et al., 2015; Martin et al., 2017), we examined learning and task performance.

## Materials and Methods

### Animals

All experiments were performed in accordance with the National Health and Medical Research Council guidelines and approved by the University of Western Australia Animal Ethics Committee (AEC 100/1453). The ephrin-A2A5^–/–^ mouse line was backcrossed onto C57BL/6J mice for >20 generations, bred from heterozygous parents, and genotyped at weaning (*n* = 32; Feldheim et al., 2000). Wild-type C57Bl/6 mice (*n* = 12) were used to clarify results obtained with ephrin-A2A5^–/–^ mice in the visual discrimination learning task. Wild-type mice were not analyzed for anatomic mapping or visuomotor head-tracking changes because we previously showed that LI-rTMS does not alter visual system topography or visuomotor head-tracking outcomes in normal wild-type mice (Rodger et al., 2012; Makowiecki et al., 2014). All mice were adult (12–52 wk old, similar average age across groups, and of either sex) at the time of testing, and thus past the critical period for visual system plasticity (Hooks and Chen, 2007). Animals were housed in 12-h day/night cycle, and food and water were provided *ad libitum* except during periods of food restriction (see Food restriction). After surgery to implant coil supports, animals were housed individually with grids removed from cages to prevent damage to supports and water gel (Necta H2O, Able Scientific) was provided for hydration. All analyses were performed blinded to treatment and task group.

### Coil supports

**Figure 1.**
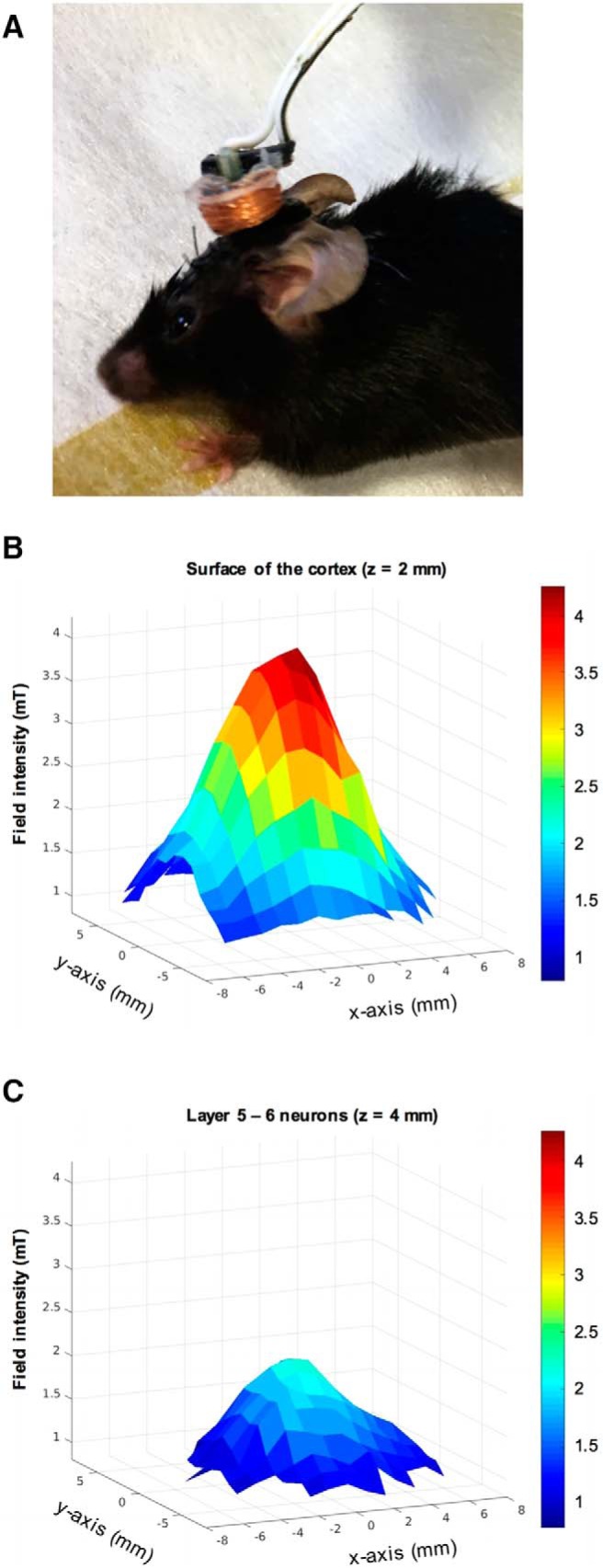
Coil attachment and magnetic field. ***A***, Photograph of an ephrin-A2A5^–/–^ mouse with a detachable coil attached to the support implant. Coil supports provided stable coil positioning for concomitant LI-rTMS or sham during the visual task. Mice did not display behavior suggestive of stress (freezing, biting, escape) after 3-d habituation to the coil and wire attachment, which did not obstruct movement. ***B***, ***C***, 3D representations of the magnetic field induced by the LI-rTMS coil. Measurements were taken on a hall device at 1-mm increments in the *x*-*y* plane and positioned 2 mm (***B***; *z* = 2) or 4 mm (***C***; *z* = 4) from the base of the coil to reflect field intensity at distances equivalent to the surface of the cortex and layer 5–6 V1 neurons, respectively.

For all mice, a coil support was attached to the skull to allow secure positioning of the coil for LI-rTMS or sham (coil switched off as no stimulation control; Fig. 1*A*). The coil support was constructed from a P20 pipette tip (Beckman-Coulter), trimmed 15 mm from the apex and fixed to a dental cement base (Paladur, Heraeus Kulzer).

To implant the coil supports, animals were deeply anaesthetized (75 mg/kg ketamine and 1 mg/kg medetomidine i.p.; Troy Laboratories), and an incision was made to expose the skull. Connective tissue on the skull surface was gently blunt-dissected. Cyanoacrylate (Uhu) was applied to the underside of the dental cement base to adhere it to the skull. The coil support fixation to the skull was then reinforced by applying further dental cement to the join between the (already set) dental cement base and the mouse skull. Excess glue and dental cement were removed, and the skin was sutured around the base of the pipette, leaving the tip accessible (Silkam, Aesculap). Pipette tips were trimmed to extend 10 mm from the surface of the skin. Anesthetic reversal (10 mg/kg atipamezole; Troy Laboratories) was injected subcutaneously.

### Food restriction

Food and hydrogel were provided *ad libitum* for 3 d after coil support implantation. Mice then commenced food restriction to 80% of free-feeding body weight (%BW) to motivate learning in the visual discrimination task. Mice in the no-task group also underwent the same food restriction protocol. Food restriction began 3 d before starting LI-rTMS or sham treatment. Mice were weighed twice daily, and food intake was modified to maintain 80–85%BW without compromising welfare.

### LI-rTMS device and coil

The custom-made coil was as described previously: 300 windings of copper wire (0.125 mm in diameter) with an inner and outer diameter of 6 and 8 mm, respectively. The coil was connected to an electromagnetic pulse generator (e-cell) programmed to deliver LI-rTMS at a biomimetic high-frequency pattern for 10 min, with trains of pulses delivered at 6–10 Hz (patent PCT/AU2007/000454, Global Energy Medicine). It has been shown that the device used at this frequency and intensity does not produce vibrations (Grehl et al., 2015) or noise audible to ephrin-A2A5^–/–^ mice (Rodger et al., 2012; Makowiecki et al., 2014; Yates et al., 2014). Maximum field intensity at the base of the coil was 12 mT (dB/dT ∼ 4.17 T/s, *z* = 0 mm, magnetic field not shown), and the estimated field strength from the cortical surface (*z* = 2 mm) to layer 5–6 (*z* = 4 mm) neurons in the primary visual cortex (V1) ranged from 3.5 to 1.5 mT, respectively (Fig. 1*B*,*C*).

### Online stimulation of freely moving mice

Mice were gently restrained by the experimenter, and coils were placed onto the plastic support and secured using an alligator clip. Mice were placed in the Y-maze (see Visual learning task), and the stimulator was switched on (LI-rTMS) or not (sham control). At the end of stimulation, the alligator clip was detached and the coil removed. Coil function was confirmed before and after each stimulation session using a Gauss meter (GM 07, Hirst Magnetics). Mice were habituated to the coils and the Y-maze environment for 5 min/d for 3 d before starting LI-rTMS.

### Visual learning task

The two-choice visual learning task comprised a custom-made Y-maze with a different stimulus displayed on a computer screen at the end of each maze arm (25 cm long). To maximally engage the visual pathway, we used moving gratings as the reward stimulus, because attention to moving targets increases neuronal responses in the superficial layers of the SC (Sefton et al., 2015). Stimuli were moving gratings (0.34 cpd, 0.008 m/s; correct target) or a gray square (incorrect target) created using Microsoft PowerPoint. The correct target was randomly presented on either end of the maze arm. At the start of each trial (i.e., initiation), mice were placed at the base of the Y-maze with the nose aligned to the midline of the box. A response was scored (manually: correct or incorrect) when mice reached midlength of an arm, and this was the time of trial completion. Correct responses were rewarded with a skewer lightly coated with peanut butter or Nutella presented to the mouse for 2 s. No reward or punishment was given for incorrect responses. During days 1–5 (habituation phase), mice were trained to associate the correct target with food rewards by gently guiding them to the target stimulus in each trial. On days 6–14, mice completed the task independently. Because we kept the duration of stimulation consistent at 10 min/d, the number of trials completed by each mouse each day was not constrained. If a trial was incomplete after 10 min, it was not recorded. The number of trials completed and response accuracy (% correct) were recorded for each training day. We analyzed median differences and distribution of groups for accuracy and cumulative number of trials from training days 6–14 (posthabituation phase). We also examined the relationship between cumulative number of correct responses as a function of number of trials.

As a control for the effects of the visual learning task, no-task mice were allowed to explore the Y-maze freely, and LI-rTMS or sham delivered as above. At random intervals during the 10-min period, mice were given food rewards and briefly removed from the Y-maze by the experimenter as a handling control.

### Locomotor activity

Locomotor activity was also assessed in ephrin-A2A5^–/–^ (LI-rTMS/sham) and wild-type (LI-rTMS/sham) mice. Mice were placed in an open field on day 14 or 15 after the start of stimulation, at least 24 h after the most recent stimulation session. The coil was switched on (or not, for sham treatment), and activity was videorecorded. The base of the open field was marked with an 8 × 8-cm grid, and the number of grid boxes crossed per minute was recorded during a 10-min period.

### Cortical injections

After 2 wk of daily LI-rTMS/sham and visual task/no-task interventions, cortical injections were performed to map the topography of corticotectal and geniculocortical projections. Ephrin-A2A5^–/–^ mice were anaesthetized as described in Coil supports and placed in a stereotaxic frame, and the coil supports were carefully removed. A small piece of skull and dura were removed to expose the left V1. Injection sites were determined visually using a landmark branch of the middle cerebral artery and confirmed using stereotaxic coordinates (Paxinos and Franklin, 2008). A nanoliter2010 (World Precision Instruments) with a micropipette was used to pressure inject two 300-nl (6 × 50-nl) injections of biotinylated dextran amine (BDA; 10,000 MW; Life Technologies) with Alexa Fluor 488 (green) and Alexa Fluor 555 (red) into lateral and medial V1, respectively, 400 μm from the surface of V1 targeting layer 5 pyramidal neurons projecting to the superficial gray layer of the SC. Injection sites were primarily within the monocular zone of V1; however, some injections were more lateral and hence likely to be within the binocular field (Sefton et al., 2015). Differences in corticotectal terminal zone (TZ) labeling based on monocular or binocular zone injections have not been reported previously and in the present study did not show any differences between red- or green-labeled TZs within the superficial gray layer of the SC, consistent with a previous rodent study (Harvey and Worthington, 1990).

### Anatomic tracing analyses

Four days after cortical injections, animals were terminally anaesthetized using sodium pentobarbitone (0.1 ml, i.p.; Lethabarb, Virbac). Mice were transcardially perfused with saline (0.9% NaCl w/v) and paraformaldehyde (4% in phosphate buffer, w/v). Whole brains were collected and postfixed in Parafresh for 24 h, cryoprotected in sucrose solution (30% in PBS w/v), and cryosectioned coronally (40 μm) in three series. One series was imaged (Nikon DS-Qi2 camera, software: NIS-Elements Basic Research) and analyzed using a Nikon e-800 fluorescent microscope to visualize anterogradely labeled TZs in the superficial gray layer of the SC, retrogradely labeled dorsal lateral geniculate nucleus (dLGN) neurons, and fluorescent injection sites in V1 layer 5. Brain regions were confirmed using adjacent Nissl-stained sections in the second series. In cases where fluorescent labeling of TZ was ambiguous (e.g., because of section damage), adjacent sections from the third series were examined with fluorescent microscopy.

### Topography of corticotectal projections

Topography of the visual field is maintained throughout the visual system (Sefton et al., 2015). For example, medial injection sites in V1 label caudal SC, whereas lateral injection sites label rostral SC (Triplett et al., 2009; Wilks et al., 2010). We specifically assessed the topographic accuracy within the horizontal axis, which is mapped from the medio-lateral axis of V1, across the rostro-caudal axis in the superficial gray layer of the SC (Harvey and Worthington, 1990; Triplett et al., 2009; Wilks et al., 2010). Medio-lateral injection site location in V1 was determined by measuring the distance from the approximate centerpoint to the medial cortical edge and expressed as a percentage of total cortical hemisphere width to normalize measures to brain size. Rostro-caudal TZ location was measured as the distance (in micrometers) from the center section of the TZ rostro-caudal span to the caudal end of the SC, expressed as a percentage of total SC span (number of sections multiplied by section thickness). Multiple TZs were scored if separated by at least one section within a single series (i.e., 120 μm) or visually distinct from an adjacent TZ.

### Topography of geniculocortical projections

Labeled dLGN neurons were analyzed to determine whether performing a visual learning task with concomitant LI-rTMS would further reduce the broad geniculocortical axonal arbors, reflected in reduced labeled dLGN cell dispersion. Cell dispersion was quantified by measuring the total area of the boundary containing the outermost labeled cells (convex-hull) in each section of one series, and multiplying by intersection distance (120 μm) to obtain convex-hull volume (in cubed micrometers). Convex-hull volumes were normalized to total dLGN volume to account for variation in dLGN size between animals. Total dLGN volume (in cubed micrometers) was measured from images of Nissl-stained sections.

To assess whether LI-rTMS affected geniculocortical topography, we also measured the area of the main cluster of labeled dLGN cells and compared LI-rTMS effects between task and no-task groups (Makowiecki et al., 2014). Focal injections in V1 retrogradely label a cluster of neighboring cells in the dLGN. Cells that are labeled but outside the main cluster have an axon branch extending beyond its appropriate location and into the injection site. Labeled cluster size (in square micrometers) in the dLGN was averaged over three sections per animal, selected from the middle span of dLGN sections with labeled cells to avoid sparse and uneven labeling toward rostral and caudal limits (Makowiecki et al., 2014). The number of labeled dLGN cells was also counted for each section in one series and multiplied by the number of series, in accordance with stereological principles.

### Visuomotor head-tracking

Visuomotor head-tracking was assessed by examining head-tracking behavior in response to moving gratings (Prusky et al., 2004; Abdeljalil et al., 2005). Mice were placed on a stationary central pedestal within a motorized optokinetic drum consisting of rotating black and white vertical gratings (1 Hz; 0.13 cpd). Light intensity was maintained at 900–1100 lux throughout testing. Mice were tested 1 d after the final LI-rTMS or sham stimulation. Mice completed 4 trials of 120 s each, alternating the grating rotation between clockwise and anticlockwise, with 30-s rest between trials. Tests were videorecorded, and the number of head-tracks per min (>1 s) was averaged across each session (Haustead et al., 2008; Rodger et al., 2012).

### Statistical analysis

Raw data were processed using Microsoft Excel and statistical analyses completed using SPSS (version 24.00, IBM) and Prism 7 (GraphPad Software).

For anatomic tracing analyses, group differences in the number of corticotectal TZs per injection were assessed by Fisher’s exact test. Linear regression was used to assess corticotectal topographical accuracy, with each injection site location (percentage in the medio-lateral axis) considered independent and plotted against TZ locations (percentage in the rostro-caudal axis). Two-way ANOVAs were used to examine the effect of treatment condition (LI-rTMS and sham) and task completion (task and no-task) on measures of retrogradely labeled dLGN neurons and visuomotor head-tracking in ephrin-A2A5^–/–^ mice.

Two-choice visual discrimination performance was assessed as accuracy and cumulative number of trials completed after the habituation phase (days 6–14). Because data did not meet assumptions of parametric statistical tests, we used two-sample Kolmogorov–Smirnov (K-S) tests to compare distributions and compared medians using Mann–Whitney *U* tests between LI-rTMS and sham for each genotype, and between ephrin-A2A5^–/–^ and wild-type mice for each treatment condition. *P*-values were Bonferroni corrected for multiple comparisons.

We used Spearman’s nonparametric bivariate correlations to assess the relationship between trials completed per day in the visual learning task and mouse weights (%BW) on each day, separately for each group, with daily %BW considered statistically independent. Two-way ANOVA was used to examine the effect of genotype (ephrin-A2A5^–/–^ and WT) and treatment condition (LI-rTMS and sham) on body weights before food restriction. A factorial between-subject ANOVA was used to compare locomotor activity between treatment conditions (LI-rTMS and sham) and genotype (ephrin-A2A5^–/–^ and wild-type). Statistical significance was set at *p* < 0.05 for all tests. Power = observed power value of the statistical test. Superscript letters listed with statistical values correspond to the statistical tests shown in Table 1.

**Table 1.** Statistical analysis

Location	Analysis	Type of test	Statistical values
Corticotectal projections			
a	Proportion of mice with >1 ectopic TZ	Fisher’s exact test	*p* = 0.99
b	TZ location (%R-C axis) against injection location (%L-M axis)	Linear regression: no-task+sham; task+sham; no-task+LI-rTMS; task+LI-rTMS	*F*_(1,9)_ = 0.799, *R*^2^ = 0.082, *p* = 0.395; *F*_(1,16)_ = 1.139, *R*^2^ = 0.076, *p* = 0.268; *F*_(1,18)_ = 8.811, *R*^2^ = 0.329, *p* = 0.008; *F*_(1,17)_ = 0.517, *R*^2^ = 0.030, *p* = 0.482
Geniculocortical projections			
c	Total dispersion volumes as % of total dLGN volume (convex-hull)	Two-way ANOVA: treatment (LI-rTMS vs sham); task completion (task vs no task); interaction (treatment × task completion)	*F*_(1,21)_ = 4.893, *p* = 0.038, power = 0.560; *F*_(1,21)_ = 0.004, *p* = 0.953, power = 0.050; *F*_(1,21)_ = 0.032, *p* = 0.860, power = 0.053
d	Average cluster areas	Two-way ANOVA: treatment (LI-rTMS vs sham); task completion (task vs no task); interaction (treatment × task completion)	*F*_(1,21)_ = 0.555, *p* = 0.465, power = 0.110; *F*_(1,21)_ = 0.214, *p* = 0.649, power = 0.073; *F*_(1,21)_ = 0.514, *p* = 0.481, power = 0.109
e	Average number of labeled dLGN neurons	Two-way ANOVA: treatment (LI-rTMS vs sham); task completion (task vs no task); interaction (treatment × task completion)	*F*_(1,21)_ = 0.740, *p* = 0.400, power = 0.130; *F*_(1,21)_ = 0.987, *p* = 0.332, power = 0.158; *F*_(1,21)_ = 1.081, *p* = 0.310, power = 0.168
f	Visual head-tracking performance	Two-way ANOVA: treatment (LI-rTMS vs sham); task completion (task vs no task); interaction (treatment × task completion)	*F*_(1,27)_ = 76.334, *p* = 0.003, power = 0.891; *F*_(1,27)_ = 1.128, **p**= 0.298, power = 0.176; *F*_(1,27)_ = 0.336, *p* = 0.550, power = 0.090
Visual learning task			
Ephrin-A2A5^–/–^ mice (LI-rTMS vs. sham)			
g	Accuracy distribution	Mann–Whitney *U* tests to compare medians. Two-sample K-S tests to compare distributions	*U* = 2190, *p* = 0.58; K-S statistic = 1.38, *p* = 0.18
h	Cumulative number of trials	Mann–Whitney *U* tests to compare medians. Two-sample K-S tests to compare distributions	*U* = 1740.5, *p* < 0.001; K-S statistic = 1.81, *p* = 0.01
Sham treatment (ephrin-A2A5^–/–^ vs WT)			
i	Accuracy distribution	Mann–Whitney *U* tests to compare medians. Two-sample K-S tests to compare distributions	*U* = 1429, *p* < 0.001; K-S statistic = 2.25, *p* < 0.001
j	Cumulative number of trials	Mann–Whitney *U* tests to compare medians. Two-sample K-S tests to compare distributions	*U* = 821, *p* < 0.001; K-S statistic = 3.01, *p* < 0.001
LI-rTMS treatment (ephrin-A2A5^–/–^ vs. WT)			
k	Accuracy distribution	Mann–Whitney *U* tests to compare medians. Two-sample K-S tests to compare distributions	*U* = 1434, *p* = 0.58; K-S statistic = 1.23, *p* = 0.39.

l	Cumulative number of trials	Mann–Whitney *U* tests to compare medians. Two-sample K-S tests to compare distributions	*U* = 1145, *p* = 0.01; K-S statistic = 1.53, *p* = 0.08.
Food restriction and visual task performance			
m	%BW and number of trials completed (sham)	Spearman’s nonparametric bivariate correlation: ephrin-A2A5^–/–^; WT	Spearman’s ρ = –0.24, *p* = 0.03; ρ = –0.03, *p* = 0.85
n	%BW and number of trials completed (LI-rTMS)	Spearman’s nonparametric bivariate correlation: ephrin-A2A5^–/–^; WT	ρ = 0.09, *p* = 0.48; ρ = 0.18, *p* = 0.18
o	Free-feeding body weight before food restriction	Two-way ANOVA: treatment (LI-rTMS vs sham); genotype (ephrin-A2A5^–/–^ vs. WT); interaction (treatment × genotype)	*F*_(1,24)_ = 0.052, *p* = 0.821, power = 0.056; *F*_(1,24)_ = 4.612, *p* = 0.042, power = 0.540; *F*_(1,24)_ = 0.571, *p* = 0.457, power = 0.112
Locomotor activity			
p	Locomotor activity in an open field	Factorial between-subjects ANOVA: treatment (LI-rTMS vs sham) main effect; genotype (task vs. no task) main effect; interaction (treatment × genotype)	*F*_(1,14)_ = 0.175, **p**= 0.682, power = 0.068; *F*_(1,14)_ = 0.055, **p**= 0.817, power = 0.056; *F*_(1,14)_ = 0.073, **p**= 0.791, power = 0.057

*P*-values Bonferroni corrected for multiple comparisons only for the visual learning task statistical analyses.

## Results

### Anatomic reorganization

To determine whether engaging in a visual learning task interacts with the effects of LI-rTMS to enhance structural reorganization in the adult ephrin-A2A5^–/–^ mouse visual system, we analyzed topographical disorder of corticotectal and geniculocortical projections.

### Corticotectal projection

**Figure 2.**
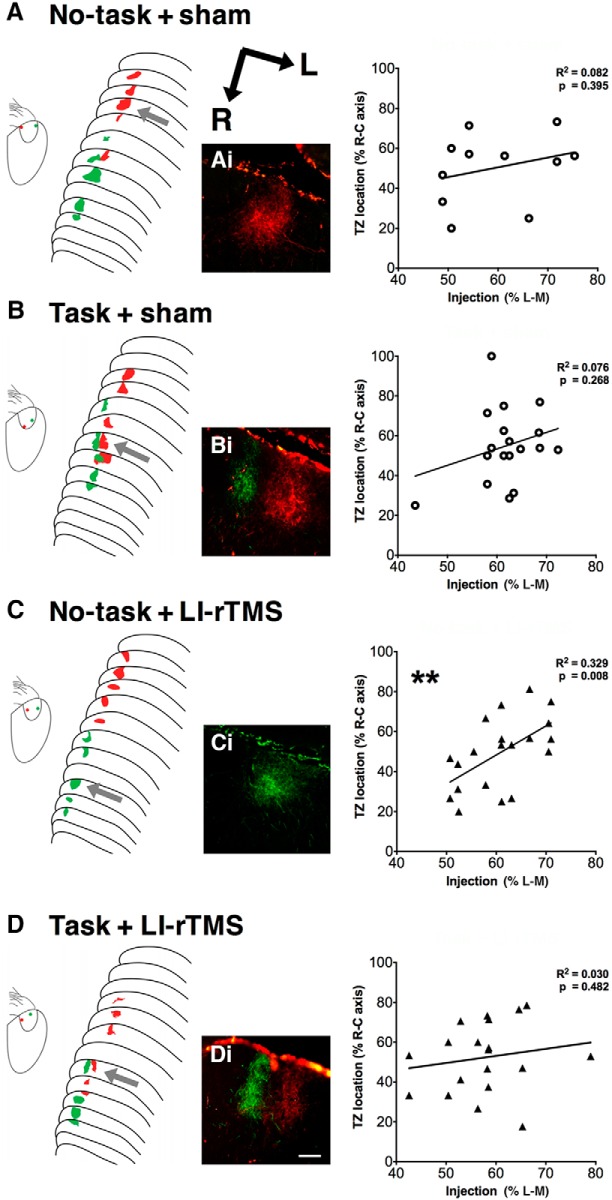
Corticotectal topography. Schematic diagrams of anterogradely labeled TZs and cortical injection locations after fluorescent medial (red) and lateral (green) V1 injections. Photomicrographs show representative TZs located in the superficial gray layer of the SC. Graphs show associated linear regression plots for each group. Twenty of 26 ephrin-A2A5^–/–^ mice showed multiple corticotectal TZs regardless of treatment and task group (Fisher’s exact test, *p* = 0.99). Injection site locations did not significantly predict TZ locations in the SC for sham-treated (***A*** and ***B***) and task + LI-rTMS (**D**) animals. ***C***, Although two green TZs were separated by 120 μm, green TZs were located at the rostral and red TZs at the caudal of the SC, with a significant relationship between injection site and TZ location. Scale bar in photomicrographs = 100 μm. **, *p* < 0.01; linear-regression analysis.

Consistent with previous reports in ephrin-A2A5^–/–^ mice, tracer injections labeled one or more TZs in the superficial gray layer of the SC (Fig. 2; Wilks et al., 2010; Makowiecki et al., 2014). Overall, 20 of 26 ephrin-A2A5^–/–^ mice (77%) showed multiple corticotectal TZs, similar to values reported previously (Rodger et al., 2012; Makowiecki et al., 2014; 78%). Two animals possessed triple TZs (both task + sham). There was no significant difference between groups in the proportion of mice with at least 1 ectopic TZ (Fisher’s exact test, *p* = 0.99^a^). The present work confirms previous findings that LI-rTMS does not reduce the incidence of ectopic TZs in the corticotectal projection of ephrin-A2A5^–/–^ mice (Makowiecki et al., 2014) and adds that engaging in a visual task, alone or in combination with LI-rTMS, has no effect on the incidence of ectopic TZs in this pathway.

Although ectopic TZs were not eliminated, LI-rTMS has been previously shown to improve the topography within the corticotectal projection, with ectopic TZs located closer to topographically appropriate positions (Makowiecki et al., 2014). We used linear regression to quantify how well the V1 injection site location predicted TZ locations (Fig. 2*A–D*), a method previously used to assess topographical accuracy within the corticotectal pathway (Triplett et al., 2009; Makowiecki et al., 2014). There was no significant linear relationship for no-task + sham mice (*F*_(1,9)_ = 0.799, *R* ^2^ = 0.082, *p* = 0.395^b^, Fig. 2*A*), consistent with the abnormal corticotectal topography previously described in ephrin-A2A5^–/–^ mice using this and other approaches (Cang et al., 2008; Triplett et al., 2009; Makowiecki et al., 2014). In addition, mice that received LI-rTMS with no task showed a moderately large positive linear relationship (no-task + LI-rTMS: *F*_(1,18)_ = 8.811, *R*^2^ = 0.329, *p* = 0.008^b^, Fig. 2*C*), suggesting that topographic order improved, consistent with a previous study showing improvement in the most disordered TZ location after LI-rTMS (Makowiecki et al., 2014). However, in ephrin-A2A5^–/–^ mice engaging in a visual task in combination with LI-rTMS or sham, topography was disordered, as revealed by injection sites not significantly predicting TZ locations (task + sham: *F*_(1,16)_ = 1.139, *R* ^2^ = 0.076, *p* = 0.268^b^, Fig. 2*B*; task + LI-rTMS: *F*_(1,17)_ = 0.517, *R* ^2^ = 0.030, *p* = 0.482^b^, Fig. 2*D*).

### Geniculocortical projection

**Figure 3.**
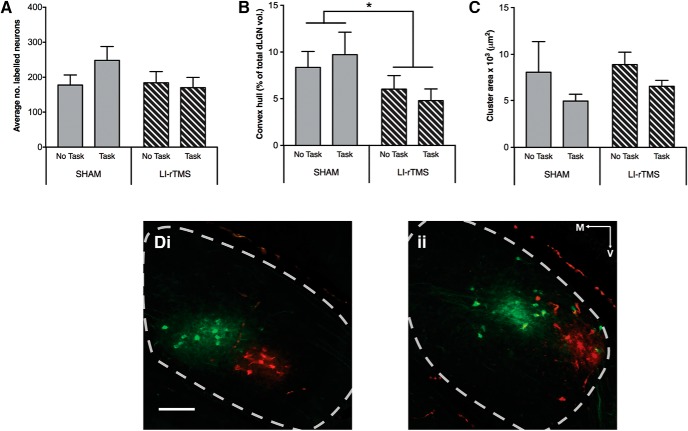
Geniculocortical projections in ephrin-A2A5^–/–^ mice. Fluorescent injections into V1 showed that the average number of retrogradely labeled dLGN neurons (***A***) was not significantly different between groups (treatment, visual task completion). However, the total dispersion volume of dLGN neurons (***B***) was significantly reduced after 14 d of LI-rTMS compared with sham-treated animals; main cluster areas (***C***) were not affected, suggesting that abnormally located geniculocortical terminals were selectively impacted by LI-rTMS, regardless of visual task completion. ***D***, Photomicrographs showing labeled dLGN neurons in LI-rTMS treated (***i***) and sham-treated (***ii***) ephrin-A2A5^–/–^ mice. Note the abnormally large dispersion of labeled red and green dLGN cells in sham-treated animals compared with LI-rTMS. Scale bar in photomicrographs = 100 μm. Error bars represent SEM. *p < 0.05; two-way ANOVA.

The average number of labeled dLGN neurons was not significantly different between groups (Fig. 3*A*). There was a significant difference in total dispersion volumes (Fig. 3*B*), but not average cluster areas (Fig. 3*C*), indicating that abnormally located dLGN axon terminals within V1 were selectively impacted by LI-rTMS (Fig. 3*D*). In contrast to the corticotectal projection, LI-rTMS–induced improvements to geniculocortical projections were not affected by combining LI-rTMS with the visual learning task (Fig. 3).

Consistent with previous results, LI-rTMS significantly reduced dispersion of labeled dLGN neurons (convex-hull volume as percentage of total dLGN volume, *F*_(1,21)_ = 4.893, *p* = 0.038, power = 0.560^c^). However, there was no significant main effect of task on cell dispersion (*F*_(1,21)_ = 0.004, *p* = 0.953, power = 0.050^c^) and no significant interaction (treatment × task: *F*_(1,21)_ = 0.032, *p* = 0.860, power = 0.053^c^). The majority of geniculocortical projections in ephrin-A2A5^–/–^ are topographically appropriate (Wilks et al., 2010; Makowiecki et al., 2014); the main cluster area represents axons appropriately projecting to the V1 injection site and was not significantly affected by LI-rTMS (*F*_(1,21)_ = 0.555, *p* = 0.465, power = 0.110^d^) or task (*F*_(1,21)_ = 0.214, *p* = 0.649, power = 0.073^d^); and there was no significant interaction (*F*_(1,21)_ = 0.514, *p* = 0.481, power = 0.109^d^). These results indicate that topographically appropriate axons (main cluster areas) were not adversely affected by treatment or task intervention. However, reduction of the abnormally broad span of projecting axons (convex-hull measurements) suggests that LI-rTMS refined geniculocortical topography.

Although there were fewer labeled dLGN neurons in LI-rTMS groups compared with sham, the comparison did not reach significance (main effect of treatment: *F*_(1,21)_ = 0.740, *p* = 0.400, power = 0.130^e^), contrasting with previous findings (Makowiecki et al., 2014). The overall number of cells labeled here was similar to numbers reported in another study (Wilks et al., 2010) but lower than in Makowiecki et al. (2014), and may have reduced sensitivity for detecting small changes. The number of labeled dLGN cells was also not affected by task completion (*F*_(1,21)_ = 0.987, *p* = 0.332, power = 0.158^e^), and there was no interaction between treatment and task completion (*F*_(1,21)_ = 1.081, *p* = 0.310, power = 0.168^e^).

### LI-rTMS improved visual head-tracking performance

**Figure 4.**
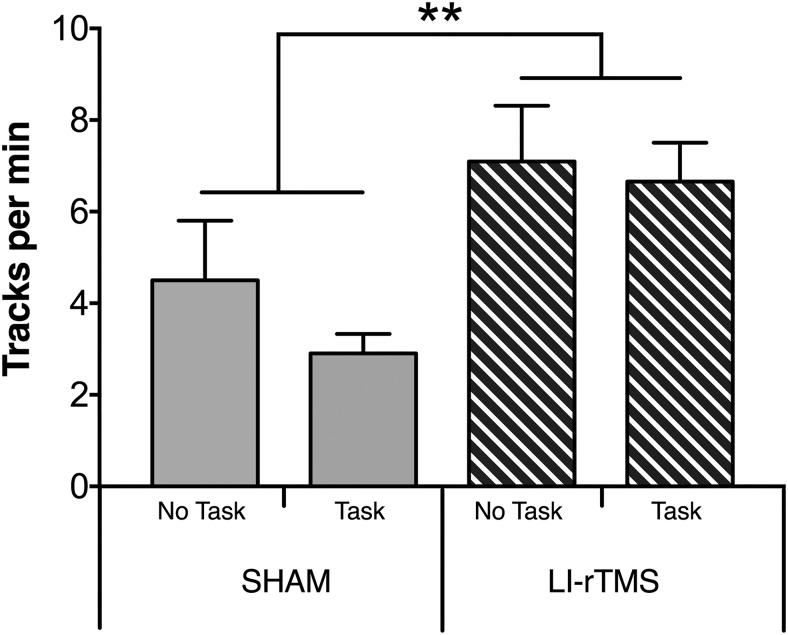
Visual head-tracking responses. Changes to visual head-tracking responses in ephrin-A2A5^–/–^ mice were assessed by averaging the number of head tracks per minute in both directions in an optokinetic drum. There was no significant difference between task groups; however, the number of head tracks was significantly higher in animals treated with LI-rTMS for 14 d compared with sham. Error bars represent SEM. **p < 0.01; two-way ANOVA.

To determine whether the neuroanatomical changes detected in the corticotectal projection after task ± LI-rTMS were associated with any change in visual function, we assessed visuomotor responses in a head-tracking test (Haustead et al., 2008; Rodger et al., 2012; Fig. 4). As previously described (Rodger et al., 2012), mice treated with LI-rTMS showed a significant improvement in the number of head-tracks (*F*_(1,27)_ = 76.334, *p* = 0.003, power = 0.891^f^). In contrast, engaging in the visual task did not improve head-tracking when applied alone (*F*_(1,27)_ = 1.128, *p* = 0.298, power = 0.176^f^) and did not alter the effects of LI-rTMS when applied in combination (interaction: *F*_(1,27)_ = 0.336, *p* = 0.550, power = 0.090^f^).

### Concomitant LI-rTMS increased number of visual learning task trials completed by Ephrin-A2A5^–/–^ mice, but not wild type

**Figure 5.**
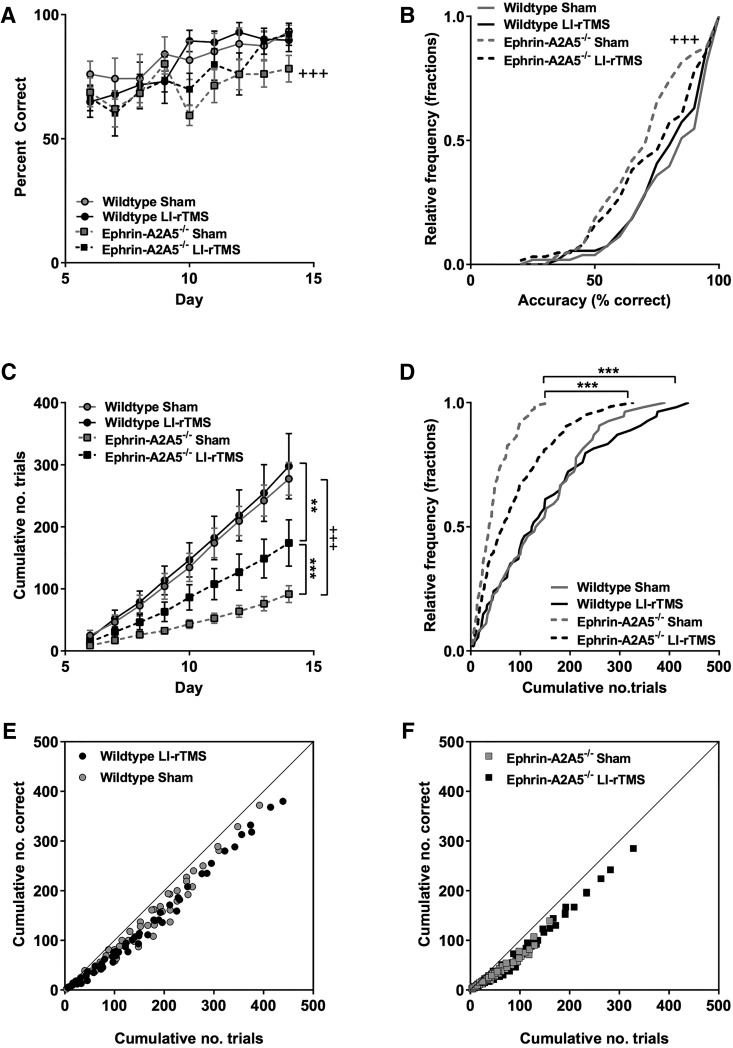
LI-rTMS during a visual discrimination task improved deficits in ephrin-A2A5^–/–^ response rates. ***A***, Group means for accuracy (percentage correct) and (***C***) cumulative number of trials completed each day (mean values shown separately for each group) for each training day of the two-choice visual discrimination task (days 6–14, after 5-d habituation). Relative frequency distributions for accuracy (***B***) and cumulative number of trials completed during the training period (***D***). ***E***, ***F***, Cumulative number of correct responses as a function of total number of trials, showing all groups close to the line of identity, indicating similar relationship between trials completed and accuracy increases over time regardless of stimulation condition in both wild-type (***E***) and ephrin-A2A5^–/–^ (***F*** mice). Error bars represent SEM. *P*-values Bonferroni corrected for multiple comparisons. **, *p* < 0.01; ***, *p* < 0.001, ephrin-A2A5^–/–^ LI-rTMS versus sham; +++, *p* < 0.001, sham ephrin-A2A5^–/–^ versus wild-type.

To confirm that mice were engaging in the visual learning task, we analyzed the accuracy and cumulative number of trials for LI-rTMS and sham in ephrin-A2A5^–/–^ mice. As expected, accuracy increased over time, indicating that animals in both LI-rTMS and sham were likely attending to the stimulus as they learned to discriminate between the target and nontarget stimulus (Fig. 5*A*). Accuracy distribution across all days was not significantly different between LI-rTMS and sham (*U* = 2190, *p* = 0.58^g^, Fig. 5*A*; K-S statistic = 1.38, *p* = 0.18^g^, Fig. 5*B*). However, cumulative number of trials was significantly greater in ephrin-A2A5^–/–^ mice receiving LI-rTMS compared with sham (*U* = 1740.5, *p* < 0.001^h^, Fig. 5*C*), and distributions were significantly different, with LI-rTMS resulting in a rightward shift in cumulative number of trials (K-S statistic = 1.81, *p* = 0.01^h^, Fig. 5*D*).

To clarify whether the behavior of ephrin-A2A5^–/–^ mice in the visual discrimination task reflected a previously undetected phenotype, we also tested wild-type mice. In wild types, LI-rTMS had no significant effect on learning task outcomes (wild-type LI-rTMS vs. sham, all *p*-values >0.05). However, compared with wild types, ephrin-A2A5^–/–^ mice receiving sham stimulation had significantly lower accuracy (*U* = 1429, *p* < 0.001^i^, Fig. 5*A*) and performed significantly fewer trials across all days (*U* = 821, *p* < 0.001^j^, Fig. 5*C*). Distribution functions of accuracy and cumulative number of trials completed over days were significantly different between wild-type and ephrin-A2A5^–/–^ mice receiving sham, with ephrin-A2A5^–/–^ mice showing significant leftward shifted distributions (accuracy: K-S statistic = 2.25, *p* < 0.001^i^; number of trials: K-S statistic = 3.01, *p* < 0.001^j^; Fig. 5*B*,*D*), suggesting a task deficit in ephin-A2A5^–/–^ mice. Interestingly, ephrin-A2A5^–/–^ mice with LI-rTMS still performed significantly fewer trials over all days compared with wild types (*U* = 1145, *p* = 0.01^l^), but accuracy was no longer significantly different (*U* = 1434, *p* = 0.58^k^) and cumulative distributions were not significantly different (accuracy: K-S statistic = 1.23, *p* = 0.39^k^; number of trials: K-S statistic = 1.53, *p* = 0.08^l^).

All groups showed a strong linear relationship between the cumulative number of correct trials over total number of trials (all *r*-values >0.98, *p*-values <0.001), indicating that the relationship between number of trials and task acquisition was not qualitatively different in ephrin-A2A5^–/–^ mice with sham compared to those with LI-rTMS or wild type. These data suggest that the deficit in ephrin-A2A5^–/–^ is due to the completion of fewer trials, delaying learning, and not a cognitive deficit per se.

Because the amount of food restriction has been linked to performance in a visual learning task (Makowiecki et al., 2012), we investigated whether there was a relationship between weight loss (i.e., hunger) and number of trials completed. Correlations between weight (as percentage of free-feeding weight) and number of trials completed each day were small for all groups (ephrin-A2A5^–/–^ mice, sham: Spearman’s ρ = –0.24, *p* = 0.03^m^; LI-rTMS: ρ = 0.09, *p* = 0.48^n^; wild-type, sham: ρ = –0.03, *p* = 0.85^m^; LI-rTMS: ρ = 0.18, *p* = 0.18^n^). Additionally, body weights before starting food restriction were not significantly different between LI-rTMS and sham groups (F_(1,24)_ = 0.052, *p* = 0.821, power = 0.06^o^). Although body weights were slightly higher in WT mice (23.90 ± 2.74 g) compared with ephrin-A2A5^–/–^ mice (21.39 ± 3.27 g; *F*_(1,24)_ = 4.612, *p* = 0.042, power = 0.540^o^), similar to previous findings in ephrin-A knockout mice (Sheleg et al., 2013, 2017), there was no significant interaction between treatment condition and genotype (*F*_(1,24)_ = 0.571, *p* = 0.457, power = 0.112^o^). Therefore, the increase in number of trials completed is unlikely to be due to differences in the level or effectiveness of food restriction in the LI-rTMS group.

To check that the increased number of trials was not just due to an overall increase in motor activity, we next examined activity in the open field test. The number of grid boxes crossed per minute was not significantly different between LI-rTMS (39.06 ± 18.54, mean ± SEM) and sham (34.79 ± 17.01; *F*_(1,14)_ = 0.175, *p* = 0.682, power = 0.068^p^) or between genotypes (*F*_(1,14)_ = 0.055, *p* = 0.817, power = 0.056^p^). There was no significant interaction between treatment condition and genotype (*F*_(1,14)_ = 0.073, *p* = 0.791, power = 0.057^p^). Therefore, the data suggest that online LI-rTMS does not have a general effect on locomotor activity, and that differences in visual learning performance were not merely due to motor hyperactivity.

## Discussion

Based on clinical reports using rTMS and exercise combinations to promote rehabilitation after brain injury (Hummel and Cohen, 2006; Wessel et al., 2015), we hypothesized that combining LI-rTMS to visual brain centers with a visual learning task that engages visual cortex would enhance LI-rTMS effects on beneficial reorganization previously observed in the abnormal mouse visual system (Makowiecki et al., 2014). However, our results suggest that combining LI-rTMS with a visual task instead prevented reorganization in the corticotectal, but not geniculocortical, pathway. In addition, LI-rTMS unexpectedly altered behavior in the visual learning task, correcting the low number of trials completed by ephrin-A2A5^–/–^ mice to near wild-type performance, raising the possibility that changes in motivation with LI-rTMS may indirectly modulate its effects on anatomic reorganization, preventing repair. Our novel online LI-rTMS approach has identified complex interactions between behavior and LI-rTMS, with implications for understanding how NBS techniques can be optimally applied in a clinical rehabilitation context.

### Corticotectal reorganization

Human studies suggest that engaging in a task simultaneously with NBS potentiates the effects of NBS on cortical excitability in the short term (Hummel and Cohen, 2006; Reis et al., 2008; Reis and Fritsch, 2011). Furthermore, rTMS as an adjunct to rehabilitative training has shown improvements to functional plasticity and specific motor performance in patients recovering from stroke (Takeuchi and Izumi, 2013; Massie et al., 2017). Such observations may be due to rTMS acting synergistically with neural activity to modulate connectivity in targeted and/or interconnected brain regions (Kastner and Ungerleider, 2000). However, our linear regression analyses (prediction of injection site location in the mediolateral axis to actual location of TZ in the rostrocaudal axis) suggest that the addition of a visual task prevented the anatomic reorganization of corticotectal connections observed in LI-rTMS–treated mice.

A possible explanation for the surprising lack of corticotectal reorganization when LI-rTMS was delivered concurrently with a visual task is that both normal and abnormal corticotectal projections were equally reinforced. Although we did not measure electrophysiological changes in our study, there is evidence that excitation of visual cortical neurons by subthreshold rTMS can sum with the neuronal activity evoked by a visual stimulus to increase response probability and visual sensitivity (Abrahamyan et al., 2011). In addition, studies in healthy human volunteers have shown that attention modulates plasticity induced by rTMS in the motor cortex (Wolters et al., 2003; Stefan et al., 2004) and visual cortex (Kamke et al., 2012, 2014) and can enhance the strengthening and suppress the weakening of neural connections representing events within the focus of attention (Kamke et al., 2012, 2014). In ephrin-A2A5^–/–^ mice, the abnormal corticotectal projections are functional (Cang et al., 2008), and the visual information they carry, even inappropriate, could have been reinforced through increased attention to the visual stimuli of the discrimination task.

A further contributor to the differences seen between task and no-task animals with LI-rTMS may be increased stress levels in task mice. Although we controlled for food-restriction and handling-induced stress in no-task + LI-rTMS animals, it is possible that completing the task itself resulted in increased stress compared with no-task animals (Meijer et al., 2007). Behavioral stress has been implicated in attenuation of synaptic plasticity (Garcia et al., 1997; Maroun and Richter-Levin, 2003), and increased stress levels in task mice may have mitigated the plastic effects of LI-rTMS. However, chronic LI-rTMS results in brain-derived neurotrophic factor upregulation (Rodger et al., 2012; Makowiecki et al., 2014), which can ameliorate stress-induced impairments to spatial learning, memory, and LTP (Radecki et al., 2005). The impact of stress on LI-rTMS effects therefore remain unclear.

We note that our results in mice that received LI-rTMS alone differ from previous findings (Makowiecki et al., 2014). In the earlier study, LI-rTMS selectively improved location of the most abnormally located TZs within mice, but when examining both appropriate and ectopic TZs together, there was no significant relationship between V1 injection location and the TZ locations in the SC. In contrast, this relationship was significant in our current study (Fig. 2), suggesting that repair by LI-rTMS may have been more effective. Differing experimental factors between the two studies might explain the discrepancy: in the present study, all mice were food restricted, which in itself, enhances synaptic plasticity in the hippocampus (Fontán-Lozano et al., 2007), and in adult rats, reinstated visual cortical plasticity capabilities normally limited to juveniles (Spolidoro et al., 2011). Thus, food restriction may have contributed to the improved outcomes observed here. Furthermore, in the current experiments, the LI-rTMS coil was secured to the mouse’s head during stimulation, ensuring a consistent location and distance from the cortex while the mouse was allowed to move freely. This approach contrasts with the previous study, in which mice received stimulation under light restraint. Because restraint stress is detrimental to plasticity (Kim and Diamond, 2002), this may have reduced efficacy of LI-rTMS; additionally, locomotion modulates V1 inhibition (Pakan et al., 2016), and combining LI-rTMS with locomotion, but not the learning task, may also have improved the efficacy of LI-rTMS–induced reorganization by enhancing baseline plasticity capability.

### Head tracking behavior

In contrast to the corticotectal outcomes, the LI-rTMS-induced improvement in visuomotor tracking was not compromised by the visual task. Visuomotor tracking relies on the SC to integrate retinal and cortical input with motor output to control the head and neck muscles (Schneider, 1969; Sefton et al., 2015). Previous studies have shown reorganization in both the corticotectal (Makowiecki et al., 2014) and retinotectal (Rodger et al., 2012) pathways after LI-rTMS, suggesting that either or both pathways could have contributed to the improvement in visuomotor tracking. However, the dissociation between visuomotor tracking improvement and structural reorganization in the corticotectal pathway we observe here suggests that it will be important in future studies to study the retinotectal projection, as well as pretectal structures, to determine whether improved topography in one or more of these pathways may be sufficient to rescue visuomotor function.

### Online LI-rTMS alters behavior in a visual learning task

The behavioral change induced by LI-rTMS in ephrin-A2A5^–/–^ mice raised the possibility that this strain might have a baseline deficit in the learning task that was rescued by the stimulation protocol. Our findings confirmed an abnormally low response rate in sham-treated ephrin-A2A5^–/–^ mice, and additionally revealed reduced accuracy compared with wild-type mice. Although both accuracy and number of trials increased in LI-rTMS–treated ephrin-A2A5^–/–^ mice, the association between cumulative trials completed and accuracy was similar in all groups. The implication is that the lower accuracy in sham ephrin-A2A5^–/–^ mice was not due to a cognitive deficit, but rather to less “practice” or learning opportunity because of their lower response rate. Our findings are consistent with previous behavioral studies of ephrin-A2A5^–/–^ mice that did not find a difference in accuracy in the visual discrimination learning task, although there were subtle differences in learning strategy in ephrin-A2^–/–^ mice (Arnall et al., 2010).

The phenotype of reduced response in ephrin-A2A5^–/–^ mice in the absence of LI-rTMS is presumably caused by altered neural circuitry in these mice. Ephrin-A2 and -A5 guidance cues are crucial for normal brain development, and mice with altered expression of these genes are known for their sensorimotor mapping phenotypes (Cang et al., 2005), raising the possibility that the lower response rate in ephrin-A2A5 mice, corrected by LI-rTMS, might be due to subtle deficits in motor activity. However, our current open field results rule out an effect of LI-rTMS on locomotion, contrasting with evidence in rats that accelerated high-intensity and high-frequency rTMS induces hyperactivity in the open field, a difference that may be due to the intensity of stimulation (El Arfani et al., 2017). A more likely explanation is that LI-rTMS may improve response rate deficits in ephrin-A2A5^–/–^ mice via modulation of the dopaminergic system, which is abnormal in ephrin-knockout mice (Cooper et al., 2009; Yates et al., 2014), and is implicated in the motivation to obtain a food reward (Berridge, 2007). Ephrin-A5^–/–^ mice have reduced dopamine concentrations in brain regions including the striatum (Sheleg et al., 2013), suggesting that low levels of dopamine may also be present in ephrin-A2A5^–/–^ mice and may explain reduced response rate in ephrin-A2A5 mice receiving sham in our study.

The possible low baseline dopamine in ephrin-A2A5^–/–^ mice suggests a mechanism for behavior rescue by LI-rTMS, because dopamine levels increased after various brain stimulation protocols in human and animal models (ELF-MF, Lee et al., 2001, Sieroń et al., 2004, Shin et al., 2007, 2011); rTMS, Strafella et al., 2003, Ohnishi et al., 2004). Acute delivery of rTMS has been shown to increase dopamine levels (Keck et al., 2002; Kanno et al., 2004), with an associated increase in expression of *c-Fos* ^+^ immunostained cells in the dorsolateral striatum (Cacace et al., 2017). These rTMS-induced changes to dopamine are likely to be mediated by the corticostriatal network rather than direct stimulation of midbrain dopaminergic neurons that project to the striatum, because neurons in the thalamus and substantia nigra remain *c-Fos*–negative after rTMS (Cacace et al., 2017). It is therefore possible that LI-rTMS increased dopamine levels in the brains of ephrin-A2A5^–/–^ mice, improving the abnormally low response rates. Consistent with this hypothesis, dopamine transporter knockdown mice, in which extracellular striatal dopamine levels are chronically elevated (Zhuang et al., 2001), had significantly higher response rates compared with wild-type mice in an instrumental learning task (Salamone and Correa, 2002; Cagniard et al., 2006; Yin et al., 2006). Further studies are required to investigate the role of dopamine in our model, including measurements of dopamine levels and using chronic delivery of D_1_- and D_2_-receptor antagonists concomitant with LI-rTMS and tasks to study motivation specifically (Shin et al., 2007).

Other brain regions may also have been affected by LI-rTMS and have contributed to the performance changes seen in LI-rTMS–treated mice in the present study. The cerebellum has been implicated as affecting motivational behaviors, with disynaptic projections to the striatum (Hoshi et al., 2005) and vice versa (Bostan et al., 2010). Lesions to the dentate nuclei of the cerebellum have been shown to impair performance in an operant conditioning task that assesses hedonic motivation for a food reward (progressive ratio breakpoint task), as well as decreasing exploratory behavior in an open field (Bauer et al., 2011). These behavioral changes were observed in the absence of any gross motor impairments or changes to anxiety levels. In addition, recent computational modeling has demonstrated complementary effects between the basal ganglia and cerebellum on goal-directed behaviors, influencing reward modulated heterosynaptic plasticity in the thalamus via dopaminergic projections (Dasgupta et al., 2014). Therefore, future exploration of online LI-rTMS effects on motivation may also investigate the contribution of on cerebello-thalamocortical loops, together with the basal ganglia, on goal-directed behaviors.

### Network effects of behavioral task may prevent V1 circuit reorganization by LI-rTMS

Because we delivered LI-rTMS simultaneously with the learning task, it is possible that LI-rTMS-induced reorganization was prevented via LI-rTMS effects on behavior, rather than a direct interaction between LI-rTMS and neural activity evoked by the task. For example, if the behavioral changes we observed with LI-rTMS were indeed accompanied by increased dopamine levels in task + LI-rTMS mice, this may have contributed to the lack of structural reorganization in the corticotectal projection: administration of D_1_-like agonists inhibits depotentiation, i.e., the ability to reverse LTP, at CA1 synapses (Otmakhova and Lisman, 1998), and may thus have prevented the shift in ectopic corticotectal projections toward more normal locations by nonselectively maintaining both appropriate and ectopic terminals, or by countering of LTD that may be required to shift ectopic corticotectal projections (Makowiecki et al., 2014). Interestingly, the dLGN receives small dopaminergic projections (Papadopoulos and Parnavelas, 1990) from the locus coeruleus (Kromer and Moore, 1980; García-Cabezas et al., 2009), potentially explaining why the visual task had no significant impact on LI-rTMS effects in this structure. The finding provides further evidence that multiple mechanisms of action are involved in LI-rTMS–induced reorganization in different relays of the visual pathway (Rodger et al., 2012; Makowiecki et al., 2014).

Finally, increased activity in the motor cortex, whether induced directly by LI-rTMS or indirectly by increased trial completion, has the potential to modulate circuitry in V1 via layer 1 neuron– and L2/L3 VIP-cell–mediated inhibitory and disinhibitory circuits (Ibrahim et al., 2016). These inhibitory circuits have been shown to be affected by high intensity (Mix et al., 2015) and potentially LI-rTMS (Tang et al., 2015), suggesting a possible mechanism for disruption of corticotectal repair observed in our study. It will therefore be important in future studies to test whether LI-rTMS delivery before (priming) or after (reinforcement/interference) the learning task might have a different outcome on reorganization.

## Conclusions

In human studies, engaging in a task simultaneously with NBS potentiates the effects of NBS on cortical excitability (Hummel and Cohen, 2006; Reis et al., 2008; Reis and Fritsch, 2011). In contrast, our results indicate that engaging in a visual task may prevent long-term beneficial reorganization induced by LI-rTMS in specific pathways within an abnormal visual network. Therefore, to deliver safe and therapeutic rehabilitation, further studies are necessary to relate the underlying cellular and molecular changes to functional outcomes after chronic administration of LI-rTMS when combined with a behavioral task.

Our results also suggest that LI-rTMS may modulate pathways involved with motivation in an abnormal system (ephrin-A2A5^–/–^ mice), perhaps via facilitation of dopaminergic neurotransmission. Chronic administration of LI-rTMS may therefore be useful not only to improve compliance in rehabilitative training, but also for patients suffering from neuropsychiatric disorders such as attention deficit disorder or major depression (Paus and Barrett, 2004). Our study highlights potentially dissociable or opposing actions of LI-rTMS: (1) increase motivation or (2) drive reorganization of abnormal circuits. In addition, we have successfully established a method to investigate the effects of online LI-rTMS on behavior in awake and freely moving mice, enabling a better understanding of the cellular, anatomic, and behavioral effects of rTMS for effective translation into the clinic.

## Synthesis

Reviewing Editor: Thomas McHugh, RIKEN Brain Science Institute Decisions are customarily a result of the Reviewing Editor and the peer reviewers coming together and discussing their recommendations until a consensus is reached. When revisions are invited, a fact-based synthesis statement explaining their decision and outlining what is needed to prepare a revision will be listed below. The following reviewer(s) agreed to reveal their identity: Hajime Hirase, Jason Triplett.

Both reviewers found the revised manuscript to be significantly improved, noting that the the re-framing of the problem and the inclusion of new data clarified the conclusions. While they agree that the manuscript will be useful to the field, they had a few remaining questions that need to be addressed prior to publication.

Comments/Questions

1. In the description of the visual learning task, it is unclear if the reward stimulus is always placed on the same side of the Y-maze. Was the grating always on the same side or was the stimulus presented randomly on either side? This is critical, since one is indicative of visual learning, while the other is indicative of spatial learning.

2. Another point of clarification is needed concerning completion of trials. The animals are allowed to freely engage in the task for 10 mins/day, so the number of trials is unconstrained. But it is unclear how the initiation and completion of trials is determined.

3. The inclusion of new thalamocortical tracing data is interesting, especially considering it appears to be distinct from the corticotectal findings, however the data would be more convincing if representative images accompanied the bar graphs.

Minor Issues

1. Line 249 - remove “topographically appropriate”

2. Lines 351-3 - This sentence seems to contradict itself. One the one hand, topography is not disrupted in the first place, so LI-rTMS doesn't affect it. On the other hand, it is, and LI-rTMS fixes it.

3. Line 546 - Add ‘The possible..’ before ‘low baseline dopamine...’
